# Data for whole and mitochondrial proteome of human embryonic stem cells

**DOI:** 10.1016/j.dib.2017.05.036

**Published:** 2017-06-12

**Authors:** Faezeh Shekari, Hossein Nezari, Yu-Ju Chen, Hossein Baharvand, Ghasem Hosseini Salekdeh

**Affiliations:** a Department of Molecular Systems Biology, Cell Science Research Center, Royan Institute for Stem Cell Biology and Technology, ACECR, Tehran, Iran; b Department of Developmental Biology, University of Science and Culture, Tehran, Iran; c Department of Stem Cells and Developmental Biology, Cell Science Research Center, Royan Institute for Stem Cell Biology and Technology, ACECR, Tehran, Iran; d Institute of Chemistry, Academia Sinica, Taipei, Taiwan; e Department of Systems Biology, Agricultural Biotechnology Research Institute of Iran, Karaj, Iran

## Abstract

The data presented here pertain to the research article entitled “Proteome Analysis of Human Embryonic Stem Cell Organelles” (Shekariet al., 2017 [1]). In the present article we endeavour to locate new proteins and pathways in human embryonic stem cells (hESCs) by mass spectrometry and bioinformatics analysis. We have analyzed total and mitochondrial proteins extracted from three biological replicates of the hESC H9 cell line according to mass spectrometry proteomics and bioinformatics investigations.

**Specifications Table**

**Table:** 

Subject area	Biology
More specific subject area	Mitochondria proteome of human embryonic stem cells (hESCs)
Type of data	Tables, figures, text files
How data was acquired	The hESCs were cultured on mouse embryonic fibroblast (MEF) cells and harvested.
	Extracted proteins from whole cell and isolated mitochondria were subjected to mass spectroscopy (Triple TOF 5600) and Western blot analysis.
Data format	Raw, filtered, analyzed
Experimental factors	No pretreatment
Experimental features	Whole proteome and mitochondria fractions of the hESC H9 cell line were subjected to mass spectrometry based identification followed by bioinformatics analysis.
Data source location	Tehran, Iran and Taipei, Taiwan
Data accessibility	The data are available with this article.

**Value of the data**

• Data present the whole proteome of the hESC H9 cell line.
• This data is the first report of the mitochondria proteome of hESCs.
• Western blot analysis and a mitochondrial proteome database search have confirmed the efficiency of the reported protocol for mitochondria isolation from hESCs. This data could enable other researchers to use the reported protocol for isolation of mitochondria.
• This data may be used to develop and enrich our knowledge about localization of proteins in mitochondria.

## 1 Data

### 1.1 Mass spectrometry based whole proteome profiling and bioinformatics analysis

A total of 470 out of 1516 proteins (Supplementary Table S1) identified in 3 replicates of hESCs (H9 cell line) were observed in only one replicate (Supplementary Fig. S2). Although most lacked transmembrane helices, approximately 60 proteins had at least two transmembranes comprised of up to 14 helices (Supplementary Table S2).

We mapped the proteins to KEGG biochemical pathways using KOBAS 3.0. The signaling components of 46 KEGG signaling pathways were found in the hESCs (Supplementary Table S3). The significantly enriched pathways included HIF-1, cGMP-dependent protein kinase (cGMP-PKG), Glucagon, Rap1, and Hippo, which highlighted the importance of these pathways in hESCs.

### 1.2 Transcription factors in the whole human embryonic stem cell (hESC) proteome profile

From the reproducibly identified proteins, 51 proteins were annotated as transcription factors according to Vaquerizas et al. [2] or AnimalTFDB [3] (Supplementary Table S4). This list included well-known TFs - signal transducer and activator of transcription 1 (STAT1), protein lin-28 homolog A (LIN28A), and spalt-like transcription factor 4 (SALL4).

### 1.3 Mass spectrometry based mitochondrial proteome profiling and bioinformatics analysis

We identified approximately 1500 proteins in three mitochondrial replicates (Supplementary Table S5). Approximately 200 proteins had at least two transmembranes of up to 15 helices. We analyzed the list of the 958 proteins found in at least two replicates with the MitoMiner 4.0 v 2016 APR database of the mitochondrial proteome (MRC Mitochondrial Biology Unit, University of Cambridge, UK; Table 1) [4]. A comparison of the identified mitochondrial proteomes with other subcellular fractions has been published in the article entitled “Proteome Analysis of Human Embryonic Stem Cell Organelles” [1].

**Table 1:** Localization of identified proteins in the mitochondrial fraction of human embryonic stem cells (hESCs) according to MitoMiner 4.0 v2016 APR. MitoMiner reported mitochondrial localization of proteins based on subcellular immunofluorescent staining results from the Human Protein Atlas, large-scale mass-spectrometry, and GFP tagging data sets as well as computational predictions of mitochondrial targeting sequences from three popular mitochondrial target sequence prediction programs: iPSORT [5], TargetP [6], and MitoProt [7].

**Mitochondria localization evidence (percentage)**	
Mito Evidence Mass-Spec Independent Studies	70.5
Mito Evidence GFP	4
Mito Evidence Mass-Spec Experiments	70.5
Mito Evidence Gene Ontology Annotation	38.5
Mito Evidence MitoCarta	33.9
Mito Evidence IMPI score (>0.7)	39.9
Mito Evidence Human Protein Atlas	23.3
Mito Targeting Seq iPSORT (>0.7)	26.3
Mito Targeting Seq MitoProt (>0.7)	31.8
Mito Targeting Seq TargetP (>0.7)	21.5
Mito Targeting Seq MitoFates (>0.7)	18.4

## 2 Experimental design, materials and methods

All materials were purchased from Sigma unless otherwise noted.

### 2.1 Human embryonic stem cell (hESC) culture and alkaline phosphatase staining

Mouse embryonic fibroblast (MEF) cells provided the feeder for the hESC culture. The hESC H9 cell line (P35-50; WiCell Research Institute, Inc., Madison, WI, USA) was cultured on mitomycin C-treated (10 μg/ml, Sigma) inactivated MEF cells (2×10^4^ cells/cm^2^) in DMEM/F-12 medium plus 20% knock-out serum replacement (Invitrogen) and 4 ng/ml of bFGF. Mechanical passaging was performed after 6–7 days, depending on confluency.

**Fig. 1:**
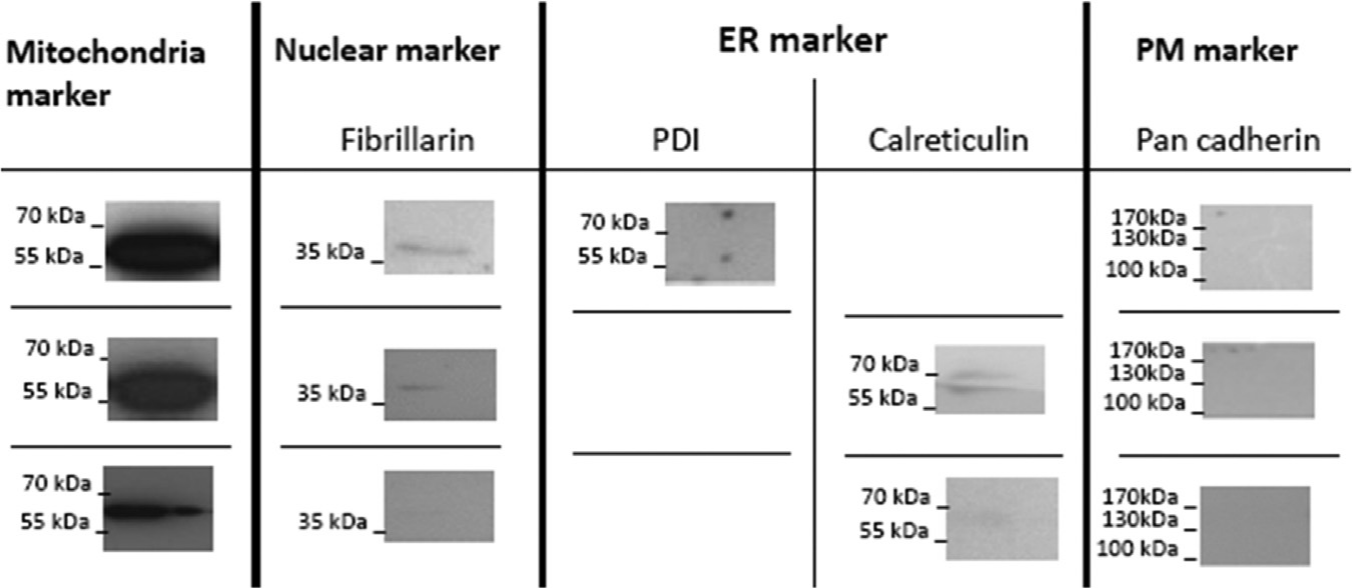
Western blot analysis of isolated mitochondria. The membranes were blotted with anti-mitochondria antibody (Abcam, UK, ab3298, 1:1000), mouse monoclonal anti-fibrillarin [38F3] antibody (Abcam, UK, ab4566, 1:2000), rabbit polyclonal anti-calreticulin antibody (Abcam, UK, ab22683, 1:1000), mouse monoclonal anti-PDI antibody (Abcam, UK, ab3672, 1:1000), and rabbit polyclonal anti-pan cadherin antibody (Abcam, UK, ab16505, 1:1000).

### 2.2 Mitochondria isolation from human embryonic stem cells (hESCs)

**Fig. 2:**
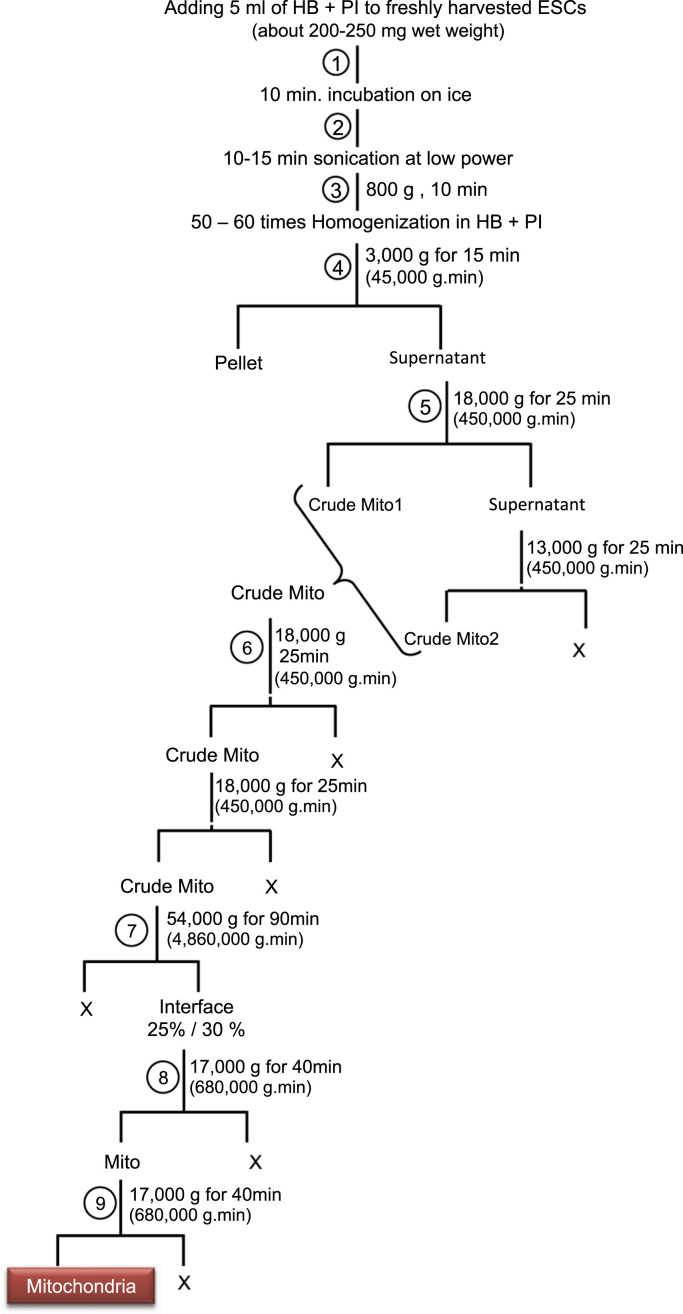
Protocol for isolation of mitochondria. The cells were homogenized in homogenization buffer (0.25 M sucrose, 10 mM HEPES, pH 7.5) that contained a protease inhibitor cocktail (Calbiochem, Germany), and subsequently sonicated for 10–15 min at low power in a Diagenode Bioruptor^®^ Sonicator. The cells were centrifuged at 800 g for 10 min. The resultant pellet was mixed with homogenization buffer and homogenized by a tight glass homogenizer until no unbroken cells remained. The supernatant was centrifuged at 3000 g for 15 min to isolate the mitochondria. The crude mitochondria fraction was pelleted by a two tandem centrifugation at 18000 g for 25 min, then washed twice with resuspending buffer that consisted of 200 mM mannitol, 50 mM sucrose, 1 mM EDTA, 0.5 mM EGTA, and 10 mM Tris HCl at pH 7.4. We prepared Nycodenz (Axis-Shield, Norway) gradients (23%, 25%, 30% and 34%) in Tris buffer that consisted of 5 mM Tris HCl (pH 7.4), 1 mM EDTA, and 0.5 mM EGTA. The mitochondria fraction was isolated in a 25/30% interface by centrifugation at 54,000 g for 90 min, after which it was pelleted and washed once by the addition of resuspending buffer.

Fig. 2 shows the protocol for mitochondrial isolation from freshly harvested hESCs.

### 2.3 Western blot analysis

A total of 10 µg of extracted protein from isolated mitochondria (based on the BCA assay) were resolved by 12% SDS-PAGE using a Mini-PROTEAN 3 electrophoresis cell (Bio-Rad), then transferred onto PVDF membrane by wet transfer in Towbin electroblotting transfer buffer (Fig. 1).

### 2.4 Gel-assisted digestion and mass spectrometry analysis

Proteins were extracted by USH buffer that contained 2 M urea and 2% SDS in 10 mM HEPES buffer. A total of 20 ug of protein was reduced and alkylated by tris (2-carboxyethyl) phosphine (TCEP) and methyl methanethiosulfonate (MMTS). Next, we added 40% acrylamide:bisacrylamide (29:1 v/v, 5:14 sample volume), 10% w/v APS (0.7:14 sample volume), and 100% TEMED (0.3:14 sample volume) directly to the sample. The sample was subsequently polymerized. The resultant gel was cut into small pieces and washed with 50% acetonitrile (ACN) in triethylammonium bicarbonate (TEABC), TEABC, 50% ACN in TEABC, 100% ACN, TEABC, and 100% ACN. The gels were completely dried, after which they underwent proteolytic digestion with trypsin (2 µg/20 µg of protein) in 25 mM TEABC for 16 h at 37 °C. Sequential extraction was performed by 25 mM TEABC, 0.1% (v/v) Trifluoroacetic acid (TFA) in water, 0.1% (v/v) TFA in ACN, and 100% ACN for peptide extraction from the gel. The extracted peptides were solubilised in 0.1% TFA, desalted with a C18 ZipTip (Millipore, UK) pipette tip, and subjected to analysis with a TripleTOF 5600 (AB SCIEX, Canada) mass spectrometer. A peptide solution was prepared by the addition of 0.1% formic acid (FA) to a concentration of 0.25 g/µl.

### 2.5 Protein identification

Peptide and protein identifications were performed using the Mascot search engine (version 2.3.02, Matrix Science). Database searching was restricted to human tryptic peptides (IPI_human_3.87; 91464 sequences) and variable modifications of human deamidated (NQ), methylthio (C), and oxidation (M). We allowed a maximum of two missed cleavages. The peptide mass tolerance was set at 10 ppm.

The Mascot engine searched the decoy database. Both the decoy score and false discovery rates were considered for identification.

## Appendix A Supplementary material

Supplementary Fig S1. Venn diagram shows the overlap between identified proteins in three replicates.

.

Supplementary Table S1. Whole proteome of human embryonic stem cells (hESCs).

.

Supplementary Table S2. Prediction result of transmembrane helices in the whole proteome of human embryonic stem cells (hESCs). TM: Transmembrane helices.

.

Supplementary Table S3. KEGG signaling pathways found in the whole proteome of human embryonic stem cells (hESCs).

.

Supplementary Table S4. Transcription factors found in the whole proteome of human embryonic stem cells (hESCs).

.

Supplementary Table S5. Proteins identified in three replicates of the mitochondrial fraction.

## References

[1] Shekari, F., H. Nezari, M.R. Larijani, C.-L. Han, H. Baharvand, Y.-J. Chen, and G.H. Salekdeh, Proteome analysis of human embryonic stem cells organelles. Journal of Proteomics, 2017. 162: p. 108-118 10.1016/j.jprot.2017.04.017 28435121

[2] Vaquerizas J.M., Kummerfeld S.K., Teichmann S.A., Luscombe N.M. (2009). A census of human transcription factors: function, expression and evolution. Nat. Rev. Genet..

[3] Zhang H.-M., Chen H., Liu W., Liu H., Gong J., Wang H., Guo A.-Y. (2012). AnimalTFDB: a comprehensive animal transcription factor database. Nucleic Acids Res..

[4] Smith A.C., Robinson A.J. (2016). MitoMiner v3.1, an update on the mitochondrial proteomics database. Nucleic Acids Res.

[5] Bannai H., Tamada Y., Maruyama O., Nakai K., Miyano S. (2002). Extensive feature detection of N-terminal protein sorting signals. Bioinformatics.

[6] Emanuelsson O., Brunak S., von Heijne G., Nielsen H. (2007). Locating proteins in the cell using TargetP, SignalP and related tools. Nat. Protoc..

[7] Claros M.G., Vincens P. (1996). Computational method to predict mitochondrially imported proteins and their targeting sequences. Eur. J Biochem..

