# Supplementary figures and images for: Data for whole and mitochondrial proteome of human embryonic stem cells
Source: Data Brief. 2017 Jun 12;13:371–6. doi: 10.1016/j.dib.2017.05.036 (PMC5480819; doi:10.1016/j.dib.2017.05.036)

**ESC repeat1**

**ESC repeat2**

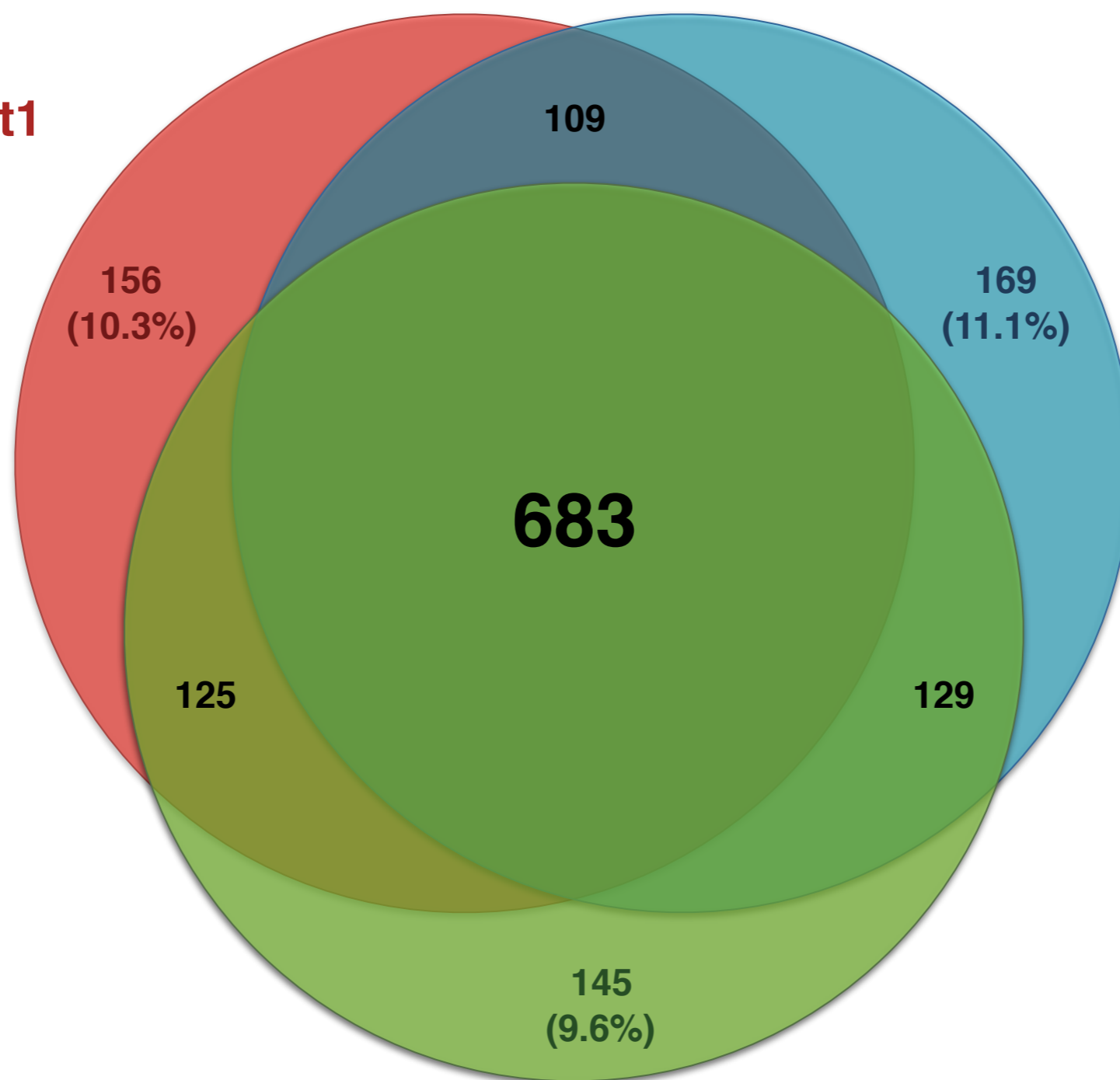

**ESC repeat3**

Supplement: Supplementary file 2 — Supplementary Fig S1. Venn diagram shows the overlap between identified proteins in three replicates. [file mmc2.pdf]
